# Evaluation of Loopamp Assay for the Diagnosis of Pulmonary Tuberculosis in Cambodia

**DOI:** 10.1155/2020/6828043

**Published:** 2020-06-01

**Authors:** Sokleaph Cheng, Sok Heng Pheng, Seiha Heng, Guy B. Marks, Anne-Laure Bañuls, Tan Eang Mao, Alexandra Kerléguer

**Affiliations:** ^1^Ministry of Health, Phnom Penh, Cambodia; ^2^Institut Pasteur du Cambodge, Phnom Penh, Cambodia; ^3^LMI “Drug Resistance in South East Asia (DRISA)”, Institut Pasteur du Cambodge and Institut de Recherche pour le Développement, Phnom Penh, Cambodia; ^4^National Center for Tuberculosis and Leprosy Control, Phnom Penh, Cambodia; ^5^Woolcock Institute of Medical Research, University of New South Wales, Sydney, Australia; ^6^MIVEGEC, UMR IRD-CNRS-Université de Montpellier, Montpellier, France

## Abstract

The Loopamp™ MTBC kit (TB-LAMP) is recommended by WHO for *Mycobacterium tuberculosis* complex detection in low-income countries with a still low drug-resistant tuberculosis (TB) rate. This study is aimed at testing its feasibility in Cambodia on sputa collected from presumptive tuberculosis patients. 499 samples were tested at a smear microscopy center and 200 at a central-level mycobacteriology laboratory. Using mycobacterial cultures as reference, TB-LAMP results were compared with those of LED fluorescent microscopy (LED-FM) and Xpert® MTB/RIF. At the microscopy center, TB-LAMP sensitivity was higher than that of LED-FM (81.5% [95% CI, 74.5-87.6] versus 69.4% [95% CI, 62.2-76.6]), but lower than that of the Xpert assay (95.5% [95% CI 92.3-98.8]). At the central-level laboratory, TB-LAMP sensitivity (92.8% [95% CI, 87.6-97.9]) was comparable to that of Xpert (90.7% [95% CI, 85.0-96.5]) using stored sample. No significant difference in terms of specificity between TB-LAMP and Xpert assays was observed in both study sites. In conclusion, our data demonstrate that TB-LAMP could be implemented at microscopy centers in Cambodia to detect TB patients. In addition, TB-LAMP can be a better choice to replace smear microscopy for rapid TB diagnosis of new presumptive TB patients, in settings with relative low prevalence of drug-resistant TB and difficulties to implement Xpert assay.

## 1. Introduction

Despite the availability of highly efficacious treatments for decades, tuberculosis remains a major public health issue worldwide. In 2017, WHO reported that 10.0 million people developed TB, mainly in developing countries, and that approximately 3.6 million (36%) were not diagnosed or notified to the national authorities [1]. The most widely used method for TB diagnosis is sputum smear microscopy. This test is simple, rapid, and inexpensive but has a relatively low sensitivity, particularly in patients with extrapulmonary TB, children, and in people with HIV [2]. Hence, the development of new diagnostic tools suitable for use in low- and middle-income countries is one of the top priorities for TB control.

Among the technologies developed and implemented over the past decade, nucleic acid amplification technologies hold the greatest promise of substantial gains in turn-around-time (compared with culture) and in sensitivity and specificity (compared with smear microscopy) [2–4]. Loop-mediated isothermal amplification (LAMP) has several advantages: no need of sophisticated instrumentation, DNA amplification of partially processed or unprocessed samples, and visual readout of the amplified products [5]. Therefore, in 2016, WHO recommended using the Loopamp™ MTBC assay (TB-LAMP) for the detection of *Mycobacterium tuberculosis* complex (MTBC) in sputum as a replacement of smear microscopy and as a follow-on test after negative smear microscopy [6]. Despite these conditional recommendations, further data are required to establish TB-LAMP usefulness in the TB diagnostic pathway in developing countries. Therefore, we assessed the feasibility and usefulness of the commercial TB-LAMP assay for the early detection and management of patients with TB at smear microscopy centers in Cambodia. Specifically, we wanted to determine the sensitivity and specificity of TB-LAMP (compared with culture as the gold standard) for the analysis of sputum specimens collected from patients with suspected pulmonary TB and tested at a smear microscopy center. We also compared TB-LAMP sensitivity and specificity with those of the Xpert® MTB/RIF (Xpert) assay and smear microscopy.

## 2. Materials and Methods

### 2.1. Study Design

We conducted two cross-sectional studies between 2013 and 2015: one at the central-level mycobacteriology laboratory using a panel of stored specimens and one in a smear microscopy center using sputum specimens prospectively collected from people undergoing TB testing in the framework of the National TB Control Program (NTP). Both study sites are located in Phnom Penh, the capital city of Cambodia. Before starting the study, the operators of TB-LAMP assay received a one-week training course administered by Eiken staffs and passed a proficiency test.

The study was approved by the National Ethics Committee for Health Research, Phnom Penh, Cambodia. Patients' informed consent was obtained.

### 2.2. Smear Microscopy Center Study

**Figure 1:**
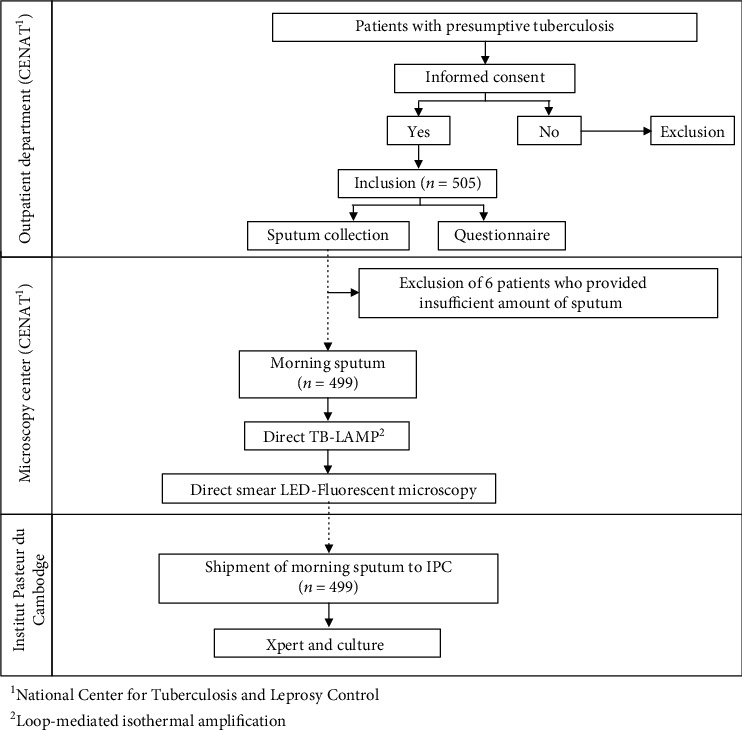
Study flowchart in smear microscopy center.

Patient enrolment was conducted at the Out-Patient Department (OPD) of TB Referral Hospital of the National Center for TB and Leprosy Control (CENAT), Phnom Penh, Cambodia (see Figure 1). Individuals aged 18 years and over with symptoms suggestive of pulmonary TB, as defined by the NTP (typically including a cough that persists for longer than two weeks and one other TB symptom, such as fever, night sweats, or recent weight loss), were eligible for the study. Patients who had been receiving any antituberculosis treatment 30 days prior to the enrolment date were excluded. Eligible patients were informed about the study by a clinician, and those who volunteered to participate were asked to sign a written informed consent and to complete a questionnaire.

Sputum samples were collected according to the NTP protocol for TB diagnosis. Each participant provided three sputum samples: a spot sputum at the time of recruitment in OPD of CENAT, a morning sputum the following day at home, and at least 1 hour later, a last spot sputum specimen when returning to OPD. It was explained to all participants in local language how to produce a good-quality sputum specimen of at least 2 ml. The spot specimen was collected under supervision. For the morning specimen, the participants received sterile, leak proof, screw-cap, and labeled sputum containers and they were asked to collect the sputum at home in the morning and to bring the specimen to CENAT the next day.

Following NTP protocol for TB diagnosis, all three sputum specimens were sent to CENAT Microscopy Center for light-emitting diode fluorescence microscopy (LED-FM). In the framework of our study, the morning sputum specimens were tested using the TB-LAMP assay by applying the same local biosafety conditions as for the preparation of microscopy. The remaining morning sputum samples were then stored at 2-8°C until shipment twice per week to the IPC Mycobacteriology Laboratory for Xpert MTB/RIF and mycobacterial culture.

#### 2.2.1. Central-Level Laboratory Evaluation

A panel of sputum samples with known smear and mycobacterial culture results was selected from the biobank of the Institut Pasteur du Cambodge (IPC) and used for the retrospective laboratory evaluation of the diagnostic performance of TB-LAMP at IPC Mycobacteriology Laboratory, which is categorized as a central-level laboratory. Two hundred sputum specimens (including 83 smear- and culture-positive, 17 smear-negative and culture-positive, and 100 smear- and culture-negative samples) that were collected between 2010 and 2012 and had been frozen at -80°C for 5 months to 2 years were included in this evaluation. Approximately 1 ml of each specimen was kept in a cryotube without the addition of chemical preservatives and without decontamination procedure prior to storage. On the specimens, TB-LAMP and Xpert MTB/RIF were performed by applying the same local biosafety conditions as for the preparation of specimens for mycobacterial culture.

### 2.3. Laboratory Procedures

#### 2.3.1. Loopamp™ MTBC Detection Kit, Eiken, Tokyo (TB-LAMP)

The TB-LAMP test was performed following the manufacturer's recommendations. DNA was extracted from the sputum samples using the Loopamp™ PURE DNA Extraction Kit. A 60 *μ*L aliquot of purulent sputum portion was mixed with the DNA extraction solution in a heating tube and incubated at 90°C in a LF-160 incubator for 5 minutes to allow bacterial cell lysis and DNA release in the solution. The DNA solution was purified through porous absorbent powder in an adsorbent tube. A 30 *μ*L aliquot of purified DNA was dispensed in the LAMP reaction tube containing the lyophilised LAMP reagents (*Bst* DNA polymerase, dNTPs, calcein, and MTBC-specific primers targeting DNA gyrase subunit B and insertion sequence *6110*). High specificity was reported by Yuki regarding the combination of primers included in the kit [7]. The dried LAMP reagents (stored in the cap of the reaction tube) were reconstituted by mixing by inversion with the DNA solution. The reaction tube was incubated at 67.0°C for 40 minutes to allow DNA amplification by the LAMP reaction. Amplified products were visually detected on the basis of their fluorescence under ultraviolet light.

#### 2.3.2. Light-Emitting Diode Fluorescence Microscopy (LED-FM)

The sputa were examined microscopically for the presence of acid-fast bacilli (AFB) by fluorescent microscopy. Direct smears were prepared for staining by Auramine O, counterstained with potassium permanganate. The slide was examined on the same day of staining at 400x magnification using an LED-based fluorescent microscope to detect yellow fluorescent rods on a dark background. The smear result was provided using a semiquantitative scale [8].

#### 2.3.3. Culture and Species Identification

At the central laboratory, 2 mL of all sputum samples underwent NALC-NaOH decontamination (1% final NaOH concentration), followed by neutralization with phosphate buffer (PB) and centrifugation [9]. Pellets were resuspended in 0.7 mL of PB and inoculated on a Löwenstein-Jensen (LJ) slant and in MGIT liquid culture tubes (Bactec MGIT; BD Microbiology Systems, Cockeysville, MD). The inoculated LJ slants were incubated at 37°C and monitored weekly for 8 weeks. Isolates from positive cultures were examined by smear microscopy using Kinyoun staining and the AccuProbe MTBC culture identification test (Gen-Probe, San Diego, CA) to identify MTBC.

#### 2.3.4. Xpert MTB/RIF (Xpert)

The Xpert assay was performed according to the manufacturer's instructions using 1 mL of untreated sputum sample or 0.5 mL of sputum pellets after a NALC-NaOH digestion method, as described above.

#### 2.3.5. Data Analysis

Using a combination of solid and liquid mycobacterial cultures as reference standard, patients were classified as being “TB-positive” when they had at least one sputum culture-positive for MTBC on solid or liquid MGIT media and as “TB-negative” in the case of negative culture or culture-positive for mycobacteria other than tuberculosis (MOTT). To assess the performance of TB-LAMP, LED-FM, and Xpert, their sensitivity was calculated as the proportion of positive tests among “TB” patients and their specificity as the proportion of negative tests among “non-TB” patients. Each value was calculated with the 95% confidence interval (95% CI). TB-LAMP and Xpert sensitivity values were also stratified according to the smear results.

TB-LAMP performance at the smear microscopy center was compared with that of the LED-FM and Xpert assays by measuring the difference in sensitivity and specificity between the tests, using a method appropriate for matched (within-subject) designs [10]. The TB-LAMP assay was considered noninferior to Xpert if the upper limit of the 95% CI of the difference for sensitivity and specificity was <5%. TB-LAMP was considered superior to smear if the lower limit of the 95% CI of the difference for sensitivity and specificity was >0. Statistical analyses were done using SAS version 9.4 (SAS Institute, Cary, NC).

## 3. Results and Discussion

### 3.1. Smear Microscopy Center Study

Between February and June 2014, 505 eligible patients with suspected TB were enrolled in the study. As six patients were excluded due to insufficient sputum volume, the study population included 499 patients (among whom 280 men). Their median age was 52 years (range 19 to 92). HIV status was unknown for most patients (471/499, 94.4%). In the 28 patients with known HIV results, three (10.7%) were HIV positive. The clinical and demographic characteristics of the study population are shown in Table 1.

Among the sputum samples of the 499 patients, 180 (36.1%) had positive mycobacterial culture on solid and/or liquid media. Most positive isolates were identified as MTBC (155/180), 23 as MOTT, and two as mixed cultures of MTBC and MOTT. Finally, 157 samples (31.5%) were classified as “TB-positive” and 342 as “TB-negative”.

The LED-FM analysis showed that 115 of the 499 samples (23%) were positive for acid-fast bacilli (AFB). Of 115 smear-positive samples, 6 (5.2%) were MOTT culture-positive. MTBC DNA was detected in 137 (27.5%) and 161 (32.3%) samples by the TB-LAMP and Xpert tests, respectively. The overall sensitivity and specificity of the LED-FM, TB-LAMP, and Xpert were calculated with 95% CI and are shown in Table 2. Using a combination of liquid and solid cultures (MGIT and LJ media) as the reference standard, the sensitivity values of TB-LAMP, Xpert, and LED-FM were 81.5% (74.5-87.6), 95.5% (92.3-98.8), and 69.4% (62.2-76.6), respectively. The sensitivity of TB-LAMP was 94.5% (90.2-98.8) in smear- and culture-positive and 52.1% (38.0-66.2) in smear-negative and culture-positive TB patients. The specificity values of TB-LAMP, Xpert, and LED-FM were 97.4% (95.7-99.1), 96.8% (94.9-98.7), and 98.2% (96.8-99.6), respectively.

Of 499 Xpert testing, 357 (71.5%) were performed on untreated sputum samples and 142 (28.5%) were performed on sputum pellets after the NALC-NaOH digestion method. The comparison of the sensitivity and specificity of Xpert performed on untreated sputum samples (96.6% [91.6-99.1] and 96.6% [93.5-98.5]) and Xpert performed on concentrated sputum (92.1% [78.6-98.3]) and 97.1% [91.8-99.4]) did not highlight any significant difference between methods.

**Figure 2:**
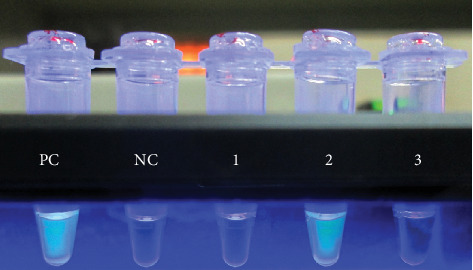
Visual detection of LAMP products under UV light. The combination of turbidity and bright green fluorescence observed in tube 2 and positive control (PC) indicated positive reactions, while a blue color without turbidity indicated a negative reaction in tubes 1, 3, and negative control (NC).

During the study, 67 series (runs) of TB-LAMP (mean number of samples tested per series: 7.6) were performed by only one operator. For each series, photos of reaction tubes exposed to ultraviolet light were captured (Figure 2). All the TB-LAMP results were read by two different individuals, first by the TB-LAMP operator and later by the laboratory supervisor using photos captured by the operator. For all the results, there was no discrepancy between the two readers. One series, which included eight samples, was needed to be repeated because of one invalid result for the positive control. All 8 samples had clear positive or negative results after repetition of TB-LAMP assay on the same sputum. No instance of contamination of the negative control or indetermination of fluorescent read-out was observed.

### 3.2. Comparison of TB-LAMP, Xpert, and LED-FM at the Smear Microscopy Center

The difference in sensitivity and specificity of TB-LAMP with that of the LED-FM and Xpert assays was calculated, and the results are shown in Table 3. The upper limit of the 95% CI for the difference in sensitivity between TB-LAMP and Xpert was 19.7%, which is above the noninferiority threshold (5%). Hence, compared with the Xpert assay sensitivity, TB-LAMP was inferior. The lower limit of the 95% CI for the difference in sensitivity between TB-LAMP and LED-FM was 5.4%, which is above the superiority threshold (0%). Hence, TB-LAMP was superior to LED-FM concerning sensitivity.

The upper limit of the 95% CI for the difference in specificity between TB-LAMP and Xpert was 0.8%, which is below the noninferiority threshold (5%). Hence, TB-LAMP was noninferior to Xpert concerning specificity. The lower limit of the 95% CI for the difference in specificity between TB-LAMP and LED-FM was -1.2%, which is below the superiority threshold (0%). Hence, TB-LAMP was not superior to LED-FM with respect to specificity.

### 3.3. Central-Level Laboratory Evaluation

In this evaluation, error results with Xpert were obtained for four samples which were excluded from the analysis, giving an indeterminate rate of 2%. Hence, only data for 196 sputum specimens (including 81 smear- and culture-positive, 16 smear-negative and culture-positive, and 99 smear- and culture-negative samples) were used to estimate TB-LAMP and Xpert sensitivity and specificity with 95% CI at the central laboratory. The results are presented in Table 2. The comparison of the sensitivity and specificity of TB-LAMP (92.8% [87.6-97.9] and 96.0% [92.1-99.8]) and Xpert (90.7% [85.0-96.5] and 97.0% [93.6-1.0]) in this setting did not highlight any significant difference between tests.

## 4. Discussion

Our findings indicate that the sensitivity of the Loopamp™ assay for TB diagnosis (TB-LAMP) is superior to that of sputum smear microscopy using the LED-fluorescent method, but inferior to that of the Xpert MTB/RIF test at the smear microscopy center. Conversely, its specificity is not superior to that of smear microscopy and is noninferior to that of the Xpert assay. However, no significant difference in sensitivity and specificity was found for TB-LAMP compared to Xpert in central-level laboratory evaluation.

In microscopy center study, TB-LAMP overall sensitivity (81.5% [95% Cl, 74.5-87.6]) and specificity (97.4% [95% Cl, 95.7-99.1]) were similar to the TB-LAMP pooled sensitivity (80.3% [95% Cl, 70.3-87.5]) and specificity (97.7% [95% Cl, 96.1-98.7]) reported by WHO [6]. TB-LAMP inferiority concerning sensitivity compared with Xpert is probably explained by the lower volume of sputum used in the test (60 *μ*L for TB-LAMP versus 1000 *μ*L for Xpert) [11]. The superiority of TB-LAMP compared with smear microscopy is highlighted in our study by the finding that approximately 50% of smear-negative samples were TB-LAMP-positive, as previously observed [3, 12–15]. In addition to the sensitivity, TB-LAMP could distinguish fairly well between MOTT and MTB; hence, it avoids inappropriate anti-TB treatment for patient with NTM infection, which is observed around 10.8% and 5.6% among patients with presumptive TB and all smear-positive cases, respectively [16]. Our results support the WHO recommendation for using the TB-LAMP test instead of smear microscopy for rapid detection of new TB patients or for follow-on testing. The inferior sensitivity (with similar specificity) of TB-LAMP compared with Xpert suggests that the latter test should be preferred. However, considering the ease of use and the low cost compared to Xpert, the TB-LAMP might be a better choice for rapid TB diagnosis of new presumptive TB patients in low- and middle-income countries (LMICs) with relative low prevalence of drug-resistant TB.

The sensitivity of TB-LAMP was reported ranging from 48% (29%-68%) in Vietnam to 100% (94%-100%) in India [6]. Among patients with bacteria-positive pulmonary TB, our data showed high sensitivity for TB-LAMP (81.5% [95% Cl, 74.5-87.6]). In two previous meta-analysis, the positivity rates of Xpert and smear microscopy were relatively high as well since the values varied from 58% to 100% and from 32% to 83%, respectively [17, 18]. Several aspects can explain these results. First, as LED-FM was performed at the CENAT microscopy center which is the reference microscopy center, the high sensitivity of smear microcopy was expected. Second, a higher positivity rate has been previously reported for the early morning sputum compared to spot sputum samples using smear microscopy and Xpert [19, 20]. Third, the selected patients had a high probability to be at an advanced state of TB since more than 80% presented a combination of symptoms such as fever, cough, chest discomfort, and weight loss and 90% had abnormal chest X-ray [21, 22]. Last, the low HIV prevalence in Cambodia can explain the high sensitivity of microscopy as well as of TB-LAMP and Xpert compared to other countries with a high HIV prevalence [1]. Besides, we cannot exclude the low performance of culture examination due to the complexity of the method [2]. However, to monitor the performance of the culture procedures, the quality indicators such as the contamination rate, artificial sputum to control cross-contamination; the systematic internal quality control; and the proficiency testing were implemented. The results of these indicators and tests were within the range defined by the European Centre for Disease Prevention and Control (ECDC). The cold chain was ensured during the transportation of the specimens until they reached culture laboratory [23].

Our result from central-level laboratory evaluation showed that TB-LAMP sensitivity and specificity were equal to those of Xpert assay. The similar performance of TB-LAMP and Xpert assays was also reported by Pham et al. in a multicenter study conducted in reference laboratories in Peru, Brazil, South Africa, and Vietnam [24]. The high sensitivity of TB-LAMP comparable to Xpert in our central-level laboratory evaluation was obtained using frozen sputum samples. The freeze and thaw processes of the samples may improve the sensitivity of TB-LAMP assay. Even if the TB-LAMP operators were well trained to take the most purulent portion of sputum for the test, due to the very low volume of sputum required (60 *μ*L), the performance of direct TB-LAMP might be affected by the viscosity of the sputum during sample splitting. It is worth noting that compared to fresh sputa, the thawed frozen sputa were relatively less viscous and easier to homogenize by a simple vortexing. Our results showed that the TB detection rate of the direct TB-LAMP was dependent on sputum quality and viscosity, as mentioned by Nguyen et al. [11]. It is noteworthy that the performance of TB-LAMP assay can reach those of Xpert when the quality and homogeneity of the sputum sample are ensured. These findings might also suggest a simple and cost-effective alternative method for TB diagnosis or for retrospective confirmation using frozen sputum sampled when the transport or processing of fresh samples is delayed, such as samples collected at remote settings in low-resource countries or used for epidemiological studies.

One limitation of the study is that follow-up for participants with TB-LAMP-positive and/or Xpert MTB/RIF-positive and bacteriologically negative results was not carried out. Mycobacterial culture is the current best available method for bacteriologically confirmed active TB patients; however, culture is not 100% sensitive due to the harmful effect of the decontamination method on mycobacteria [2]. The use of an imperfect reference standard method will have a more deleterious effect on assay with a higher specificity [24].

Another limitation is that the smear microscopy center where the study was carried out is the reference microscopy center in the country. In this reference microscopy center, the routine sputum smears are prepared in a biosafety cabinet because of the high number of sputum samples per day compared to other microscopy centers in the country. Therefore, the DNA extraction of samples for TB-LAMP assay was also performed in a biosafety cabinet. This may explain the relative low number of false positive results and the low rate of contaminations for the smear microscopy and TB-LAMP assays. However, the technician who performed TB-LAMP assay at the microscopy center has no experience on a molecular method or other laboratory method than microscopy and only received one-week training to perform the test. Further studies in microscopy centers at peripheral levels are recommended to confirm the expected benefits.

In conclusion, our findings suggest that the TB-LAMP assay can be implemented in smear microscopy centers in Cambodia and can rapidly and accurately detect patients with TB using sputum samples, with a sensitivity of 81.5% and a specificity of 97.4%. In addition, TB-LAMP sensitivity is superior to that of LED-fluorescent microscopy and requires similar infrastructure as smear microscopy. Therefore, TB-LAMP assay can be a better choice to replace smear microscopy for rapid TB diagnosis of new presumptive TB patients, in settings with a relatively low prevalence of drug-resistant TB and difficulties to implement Xpert assay.

## Figures and Tables

**Table 1:** Demographic and clinical features of the study population and culture-confirmed tuberculosis cases in smear microscopy center study.

Characteristics		Study population N (%)	Culture-confirmed TB cases N (%)
Age (year)	Total	499	157
	18-24	36 (7.2)	16 (10.2)
	25-34	83 (16.6)	31 (19.7)
	35-44	67 (13.4)	26 (16.6)
	45-54	94 (18.8)	33 (21.0)
	55-64	98 (19.6)	26 (16.6)
	≥ 65	121 (24.2)	25 (15.9)

Sex	Total	499	157
	Men	280 (56.1)	103 (65.6)
	Women	219 (43.9)	54 (34.4)

Symptoms	Total	499	157
	Fever	497 (99.6)	156 (98.1)
	Cough	487 (97.6)	154 (96.9)
	Chest discomfort	463 (92.8)	143 (89.9)
	Weight loss	453 (90.8)	150 (94.3)
	Breathlessness	98 (19.6)	35 (22.0)
	Hemoptysis	63 (12.6)	23 (14.5)

Abnormal chest X-ray		421/426 (98.8)	141/141 (100)

Previous treatment for TB disease		129/482 (26.8)	18/154 (11.7)

TB contact		74/496 (14.8)	13/156 (8.3)

**Table 2:** Sensitivity and specificity of LED-fluorescent microscopy, TB-LAMP, and Xpert MTB/RIF.

Test	Culture result		Sensitivity among culture-positive MTB			Specificity % (95% CI)
	MTB-positive (*n* = 157) positive/negative	MTB-negative (*n* = 342) positive/negative	Smear-positive % (95% CI)	Smear-negative % (95% CI)	Overall % (95% CI)	
Smear microscopy center						
Microscopy	109/48	6/336	—	—	69.4 (62.2-76.6)	98.2 (96.8-99.6)
TB-LAMP	128/29	9/333	94.5 (90.2-98.8)	52.1 (38.0-66.2)	81.5 (74.5-87.6)	97.4 (95.7-99.1)
Xpert	150/7	11/331	99.1 (93.6-99.9)	87.5 (74.2-94.4)	95.5 (92.3-98.8)	96.8 (94.9-98.7)
Central laboratory						
TB-LAMP	90/7	4/95	100	56.3 (29.8-79.6)	92.8 (87.6-97.9)	96.0 (92.1-99.8)
Xpert	88/9	3/96	100	43.8 (20.4-70.2)	90.7 (85.0-96.5)	97.0 (93.6-1.0)

**Table 3:** Matched comparison between TB-LAMP performed at the smear microscopy center and the Xpert and LED-fluorescent microscopy.

Comparison	Difference (%)	Lower 95% confidence limit (%)	Upper 95% confidence limit (%)
Sensitivity			
TB-LAMP versus Xpert	14.0	8.3	19.7
TB-LAMP versus microscopy	12.1	5.4	18.8
Specificity			
TB-LAMP versus Xpert	-0.6	-2.0	0.8
TB-LAMP versus microscopy	0.9	-1.2	2.9

## Data Availability

The data used to support the findings of this study are available from the corresponding author upon request.
